# Orthodontic Rubber Band-Assisted Endoscopic Submucosal Dissection: An Efficient Method for Treating Superficial Colorectal Tumors

**DOI:** 10.1155/2022/2835258

**Published:** 2022-01-30

**Authors:** Linfu Zheng, Wen Wang, Dazhou Li, Junguo Chen, Longping Chen, Rong Wang, Chuanshen Jiang, Guanpo Zhang, Yaping Hou, Jin Zheng, Yang Bai

**Affiliations:** ^1^Guangdong Provincial Key Laboratory of Gastroenterology, Department of Gastroenterology, Institute of Gastroenterology of Guangdong Province, Nanfang Hospital, Southern Medical University, Guangzhou 510515, China; ^2^Department of Gastroenterology, Fuzhou General Clinical Medical College of Fujian Medical University, Fuzhou 350025, China; ^3^Department of Gastroenterology, 900th Hospital of People's Liberation Army, Fuzhou 350025, China; ^4^Department of Gastroenterology, Oriental Hospital Affiliated to Xiamen University, Fuzhou 350025, China

## Abstract

**Background:**

Colorectal endoscopic submucosal dissection (ESD) is a complex operation. Effective traction is crucial. We have successfully used an orthodontic rubber band (ORB) combined with the clip traction method to assist ESD (ORB-ESD). The aim of this retrospective study is to describe the method and to compare the efficacy and safety of ORB-ESD versus conventional ESD in the treatment of superficial colorectal tumors.

**Methods:**

We retrospectively analyzed the data of patients with superficial colorectal tumor (with diameter ≥ 20 mm) who received either ORB-ESD (*n* = 34) or conventional ESD (*n* = 90) between January 2019 and September 2020. Propensity score matching (PSM) was used to match the clinical data of 31 pairs of patients in each group.

**Results:**

Operation time was significantly shorter for ORB-ESD than for conventional ESD (34.5 minutes vs. 56 minutes, *P* ≤ 0.001). In the propensity-matched cohorts, the operation time remained significantly shorter in the ORB-ESD patients (35 minutes vs. 50 minutes, *P* = 0.001). Postoperative adverse events, en bloc resection rate, and R0 resection rate were comparable between the two groups (*P* > 0.05), both before and after propensity score matching. In the ORB subgroup analysis, the trainee and expert ESD operation times were similar (37 (26–53) vs. 33.5 (26–37) minutes, respectively; *P* = 0.274).

**Conclusion:**

ORB-ESD appears to be an effective technique for ESD of colorectal cancer. Our findings need to be confirmed in large prospective multicenter studies.

## 1. Introduction

Endoscopic submucosal dissection (ESD) is widely used for the treatment of superficial gastrointestinal lesions. Good exposure of the operative field is of crucial importance during ESD. Effective traction, without undue tension on diseased tissue, makes it easier for the surgeon to identify submucosal vessels and define the direction of incision, minimize the risk of bleeding and perforation, and achieve complete resection of the lesion [1–3]. New methods to improve traction during ESD continue to be introduced; currently available methods include the sinker-assisted traction [4], clip-with-line traction [5], loops-attached rubber band traction [6], Sakamoto-Osada (S-O) clip traction [7], pocket-creation traction [8], medical ring [9] or rubber band with clips [10], and magnetic anchor traction [2]. All have their own advantages and disadvantages. Some traction methods are limited by their complexity or cost. It should be noted that colorectal ESD is particularly complex, and traction methods that are effective in the esophagus and stomach may not be suitable for use in the colorectal tract. Therefore, the search continues for a simple, economical, safe, and effective traction method for use during colorectal ESD.

In our center, we have successfully used the combination of orthodontic rubber band (ORB) and clip to achieve traction during colorectal ESD. ORB, which is widely used in orthodontics, is made of natural rubber; it has good elasticity and is available in different diameters. Importantly, it can be passed through the working channel of the endoscope directly, without the need for withdrawal and reinsertion of the colonoscope. The aim of this study is to describe the technique and to retrospectively compare the efficacy and safety of ORB-ESD versus conventional ESD in the treatment of colorectal tumors.

## 2. Methods

### 2.1. Patients

**Figure 1:**
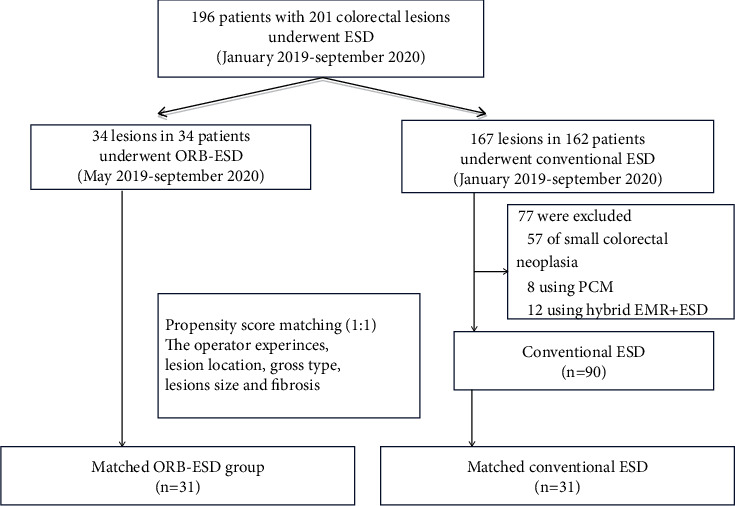
Study flowchart.

Patients with superficial colorectal tumors (with maximum lesion diameter ≥ 20 mm) who were treated by ORB-ESD or conventional ESD at the Endoscope Center of the 900th Hospital of the PLA from January 2019 to September 2020 were eligible for inclusion in this retrospective study. During the study period, 196 patients, with a total of 201 colorectal lesions, underwent ESD. From among these 196 patients, 124 patients met the eligibility criteria of this study: 34 patients received ORB-ESD and 90 patients received conventional ESD (Figure 1). The data of these patients were extracted from the case records for analysis.

This study was conducted in conformance with the tenets of the Helsinki Declaration and was approved by the Ethics Committee of the 900th Hospital of the PLA. The need for obtaining patients' consent for publication was waived as this was a retrospective analysis of anonymized clinical data.

### 2.2. ESD Procedure

All ESD operations were performed using a single-channel endoscope with a water-jet system (GIF-Q260J/PCF-Q260JI; Olympus Co., Tokyo, Japan), transparent hood (D-201-11804; Olympus Co., Tokyo, Japan), and electrosurgical generator (VIO200S; Elektromedizin Gmbh, Tubingen, Germany). Insufflation was with carbon dioxide. Submucosal injection of sodium hyaluronate mixed with 1 : 100000 adrenaline and methylene blue was performed using an NM-4U injection needle (Olympus Co., Tokyo, Japan). Dual Knife (Olympus Co., Tokyo, Japan) was used for incising the periphery of the focus and for submucosal exfoliation. An opening-and-closing clip (Micro-Tech (Nanjing) Co., Nanjing, China) was used to connect the ORB for traction, and a snare was used for removing the clip fixed on the bowel wall.

### 2.3. ORB Combined with the Clip Traction Method

**Figure 2:**
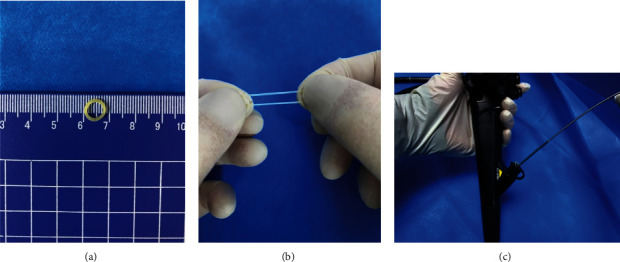
Diagram showing the application of an orthodontic rubber band (ORB): (a) the inner diameter of the ORB; (b) good elasticity of the ORB; (c) an ORB passed through the working channel of the endoscope.

**Figure 3:**
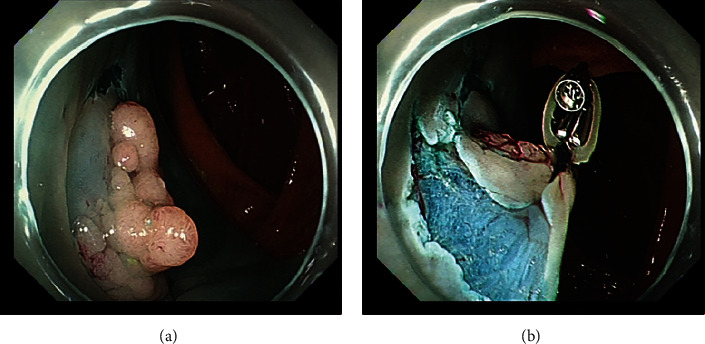
(a) An ascending colon lesion with the mucosal incision; (b) good traction effect.

**Figure 4:**
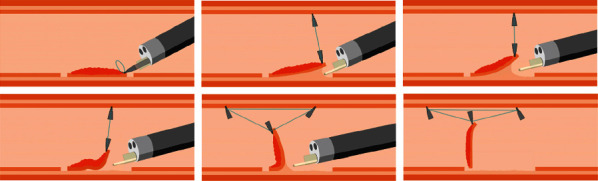
The traction effect might decrease as dissection progresses during endoscopic submucosal dissection. In such cases, an additional clip can be applied to strengthen the traction.

ORBs are available with different inner diameters (3.16 mm, 4.7 mm, 6.35 mm, 7.9 mm, and 9.5 mm). Based on our experience with conventional ESD, we use the 6.35 mm diameter ORB, combined with clip, for traction during ORB-ESD. After the circumferential incision around the lesion is made with Dual Knife, the colonoscope is not withdrawn. The ORB, with the clip applied on one side, is passed through the working channel of the endoscope into the intestinal cavity (Figure 2). The clip attached to the ORB is opened and fixed to the distal edge of the lesion. Another clip is passed through the endoscope, attached to the other side of the ORB, and then fixed to the normal mucosa opposite the lesion. Thus, effective traction is exerted during the ESD (Figure 3). If, during the process of dissection, the traction effect is weakened and the operative field is not satisfactorily exposed, another clip can be passed, clamped to the side of the ORB, and then fixed to opposite mucosa to apply traction in the required direction. In this way, the submucosa can be exposed well again to ensure that the dissection can be carried out smoothly (Figure 4). After the ESD is completed, a snare is used to remove the clip fixed on the bowel wall.

### 2.4. Outcomes

The primary outcome in this study was the operation time, i.e., the time (in minutes) from submucosal injection to complete resection of the lesion. The secondary outcomes were the incidence of adverse events related to ESD (postoperative bleeding and perforation), en bloc resection rate, R0 resection rate, and incidence of damage to the specimen. Postoperative bleeding was defined as hematochezia within 2 weeks after ESD, requiring endoscopic intervention for hemostasis. Perforation was defined as the presence of mesenteric fat within the intestinal lumen during ESD or the presence of free gas in the abdominal cavity on postoperative radiographs or CT. Damage to the specimen by the traction device was defined as damage/destruction of the resected lesion due to ORB traction or application/removal of the clip.

### 2.5. Propensity Score Matching (PSM)

Outcomes in ESD may be influenced by several factors. We therefore performed PSM to match for operator experience (nonexpert (i.e., <50 cases of colorectal ESD performed) vs. expert (i.e., >300 cases of colorectal ESD performed)) [11], location of colorectal lesion (rectum, left colon, and right colon), gross type (superficial and polypoid), lesion size, and presence of fibrosis. The caliper range was set to 0.2 with a 1 : 1 ratio for both the ORB-ESD group and the conventional ESD group.

### 2.6. Statistical Analysis

SPSS 22.0 (IBM Corp., Armonk, NY, USA) was used for statistical analysis. Continuous variables were summarized as medians (interquartile ranges) and compared using the Mann-Whitney *U* test. Categorical variables were summarized as percentages and compared using the chi-square test or Fisher exact test. *P* < 0.05 was considered statistically significant.

## 3. Results

While 34 patients underwent ORB-ESD, 90 patients underwent conventional ESD.

Superficial lesions were significantly more common in the ORB-ESD group (*P* ≤ 0.001). Operation time was significantly shorter in the ORB-ESD group than in the conventional ESD group (34.5 (26–42) vs. 56 (44–66) minutes, *P* ≤ 0.001). Mean age, sex distribution, lesion location, lesion size, proportion of surgeries performed by experts, and proportion of cases with fibrosis were not significantly different between the two groups (Table 1). The en bloc resection rate was 100% in both groups. The R0 resection rate was 97.1% in the ORB-ESD group (1 case of positive lateral margin) vs. 90.0% in the conventional ESD group (8 cases of positive lateral margin and 1 case of positive basal margin with deep invasive submucosal carcinoma (SM2)). In the ORB-ESD group, there was one case of postoperative bleeding and one case of perforation; in the conventional ESD group, there were four cases of postoperative bleeding and two cases of perforation. Endoscopic hemostasis and endoscopic perforation closure were performed successfully in both groups. The adverse event rate, en bloc resection rate, R0 resection rate, and histology were not significantly different between the two groups (*P* > 0.05 for all; Table 1).

In the ORB-ESD group (*n* = 34), the trainee and expert ESD operation times were similar (37 (26–53) vs. 33.5 (26–37) minutes, respectively; *P* = 0.274; Table 2). Five patients needed multidirectional traction in the ORB-ESD group (with the use of three clips in each case: 1 clip for fixation and 2 for traction). The integrity of the specimen was not damaged by the traction in any of the 34 patients.

### 3.1. PSM Analysis

For PSM analysis, 31 patients in the ORB-ESD group were successfully matched with 31 patients in the conventional ESD group. Baseline characteristics were comparable between the matched groups (Table 3). Operation time remained significantly shorter in ORB-ESD patients than in conventional ESD patients (35 (26–42) vs. 50 (42–64) minutes, respectively; *P* = 0.001).

## 4. Discussion

In 2005, Saito et al. reported that sinker-assisted ESD could ensure good exposure of the submucosa and greatly facilitate ESD [4]. The drawbacks of the sinker1-assisted method are the need for special equipment and for the withdrawal of the endoscope to install the traction device. The procedure is time-consuming, and sometimes, the patient's position has to be changed to expose the submucosa. Nevertheless, many subsequently introduced traction methods were based on the sinker-assisted traction method.

Commonly used traction methods during colorectal ESD include S-O clip traction [7, 12, 13], magnetic bead traction [14], and pocket-creation traction [8, 15–17]. S-O clip traction and magnetic bead traction are limited by the need for special devices, and magnetic bead traction by the need for withdrawal of the endoscope to install the traction device. During treatment of lesions in the right colon, repeated entry and exit of the colonoscope can cause colorectal spasm and increase the difficulty of the operation. There are several problems associated with the pocket-creation method also; for example, if the pocket entrance incision is too long, endoscope stability is poor and, if the incision is too short, the endoscope may not be able to enter the submucosa.

The ideal method for colorectal ESD traction should be simple, economical, convenient, effective, and easily mastered. There should be no need for complex equipment and accessories. Our method of ORB combined with clip meets the above requirements. First, ORB, which is widely used in orthodontics, is easily available. Its excellent elasticity ensures good traction. Second, it is inexpensive. Whereas the multiloop traction device recently reported by Suzuki et al. [18] costs about 20 cents (RMB 1.49 Yuan), 100 ORBs cost only 21 cents (RMB 1.38 Yuan). Third, the procedure is simple and easily mastered. There is no need to withdraw the endoscope, and so the method may be especially suitable for ESD in the right colon. Fourth, the good traction effect with this method can facilitate ESD, as demonstrated by the significantly shorter procedure time for ORB-ESD than for conventional ESD in this study. Factors such as operator experience; lesion location, shape, and size; and presence of fibrosis can affect procedure time, but operation time remained significantly shorter in ORD-ESD patients even in propensity score-matched analysis.

According to literature, postoperative bleeding and perforation occur in 1.5%-11.9% [19] and 1.4%-10.4% [20] of patients, respectively, after colorectal ESD. In our study, the postoperative bleeding rate and perforation rate were both 2.9% in ORB-ESD patients, versus 4.4% and 2.2%, respectively, in conventional ESD patients. The differences between the groups were not statistically significant, probably because of the small sample size. The R0 resection rate was higher in the ORB-ESD group than in the ESD group (97.1% vs. 90%) though, again, the difference was not statistically significant. It is also worth noting that, in the ORB-ESD group, the integrity of the specimen was not affected by the application or removal of the clip.

Furthermore, some scholars used a double clip and rubber band during colon ESD operation to improve surgical efficiency [21, 22]. It was reported that the countertraction of the rubber band was maintained by injecting air into the intestinal lumen [22]. Sometimes, the traction gradually weakens as the dissection of large lesion progresses. Excessive insufflation could lead to difficulties in colonoscopy, particularly for right colon lesions, cause premature fall off of the clip, and even cause complications such as abdominal distention and perforation. The traction effect decreased during the procedure in five of the 34 cases in the ORB-ESD group. However, application of an extra clip for multidirectional traction effectively achieved good exposure of the submucosa again. Thus, during ORB-ESD, additional clips can be applied and multidirectional traction was implemented whenever necessary to improve exposure. We compared the operation time between the trainees and experts in an ORB-ESD subgroup analysis. Although the operation time of the trainee group was numerically longer, the difference between the subgroups was statistically insignificant (37 (26–53) vs. 33.5 (26–37) minutes, *P* = 0.274). Therefore, the use of the ORB traction technology might benefit trainees during training in ESD. However, we need to draw some attention to the operation when a combination of the ORB and clip traction is used. The clip occlusion of normal intestinal mucosa should be moderate when an ORB is used with a clip. Too little occlusion of the intestinal mucosa might cause the clip to fall off during aeration, while too much occlusion might cause excessive damage to normal tissue during clip removal. Therefore, the wound surface should be observed after using a snare to remove a clip fixed on the normal intestinal wall.

This study has some limitations. First, this is a single-center retrospective study, and a selection bias is likely. However, we performed propensity score matching to reduce confounding by known factors. Second, the sample size was small, and there was no follow-up. Large multicenter randomized controlled trials are needed to confirm the superiority of the technique of ORB-ESD.

In conclusion, ORB combined with clip is an effective, simple, and economical traction technique for use during colorectal ESD. The method appears to be worthy of wide adoption.

## Figures and Tables

**Table 1:** Baseline patient characteristics and treatment outcomes in the entire cohort before propensity score matching (PSM).

	ORB-ESD group (*n* = 34)	Conventional ESD group (*n* = 90)	*P* value
Age (years)	55 (50–65)	57 (52–66)	0.466^W^
Sex, *n* (%)			0.302^C^
Male	18 (52.9)	58 (64.4)	
Female	16 (47.1)	32 (35.6)	
Gross type, *n* (%)			≤0.001^C^
Polypoid	4 (11.8)	42 (46.7)	
Superficial	30 (88.2)	48 (53.3)	
Lesion location, *n* (%)			0.093^C^
Right colon	16 (47.1)	24 (26.7)	
Left colon	6 (17.6)	25 (27.8)	
Rectum	12 (35.3)	41 (45.6)	
Lesion size (mm)	20 (20–27)	21 (20–30)	0.416^W^
Procedure time (min)	34.5 (26–42)	56 (44–66)	≤0.001^W^
Fibrosis, *n* (%)	3 (8.8)	3 (3.3)	0.344^F^
Operator experiences, *n* (%)			1^C^
Expert	14 (41.2)	38 (42.2)	
Trainee	20 (58.8)	52 (57.8)	
Complications, *n* (%)			1^F^
Delayed bleeding	1 (2.9)	4 (4.4)	
Perforation	1 (2.9)	2 (2.2)	
En bloc resection, *n* (%)	34 (100)	90 (100)	1^F^
R0 resection, *n* (%)	33 (97.1)	81 (90.0)	0.283^F^
Histology			0.275^F^
Mucosal lesion	30 (88.2)	82 (91.1)	
SM1	2 (5.9)	7 (7.8)	
SM2	2 (5.9)	1 (1.1)	

ORB: orthodontic rubber band; ESD: endoscopic submucosal dissection; SM1: superficial invasive submucosal carcinoma (<1000 *μ*m); SM2: deep invasive submucosal carcinoma (≥1000 *μ*m). ^W^Mann-Whitney *U* test, ^C^chi-squared test, ^F^Fisher's exact test.

**Table 2:** Comparison of surgical times between trainees and experts in the ORB group.

	Expert (*n* = 14)	Trainee (*n* = 20)	*P* value
Procedure time (min)	33.5 (26–37)	37 (26–53)	0.274^W^

^W^Mann-Whitney *U* test. ORB: orthodontic rubber band.

**Table 3:** Variables included in the propensity score matching, and treatment outcomes in the matched groups.

	ORB-ESD group (*n* = 31)	Conventional ESD group (*n* = 31)	*P* value
Gross type, *n* (%)			1^F^
Polypoid	4 (12.9)	5 (16.1)	
Superficial	27 (87.1)	26 (83.9)	
Lesion location, *n* (%)			0.848^C^
Right colon	15 (48.4)	13 (41.9)	
Left colon	5 (16.1)	7 (22.6)	
Rectum	11 (35.5)	11 (35.5)	
Lesion size (mm)	20 (20–27.5)	21 (20–25)	0.988^W^
Procedure time (min)	35 (26–42)	50 (42–64)	0.001^W^
Fibrosis, *n* (%)	0 (0)	1 (3.2)	0.344^F^
Operator experiences, *n* (%)			1^C^
Expert	12 (38.7)	13 (41.9)	
Trainee	19 (61.3)	18 (58.1)	
Complications, *n* (%)			1^F^
Delayed bleeding	1 (3.2)	0 (0)	
Perforation	1 (3.2)	1 (3.2)	
En bloc resection, *n* (%)	31 (100)	31 (100)	1^F^
R0 resection, *n* (%)	30 (96.8)	29 (93.5)	1^F^

ORB: orthodontic rubber band; ESD: endoscopic submucosal dissection. ^F^Fisher's exact test, ^C^chi-squared test, ^W^Mann-Whitney *U* test.

## Data Availability

The datasets used and analyzed during the current study are available from the corresponding author on reasonable request.
